# Incidental biliary adenofibroma with dysplastic features

**DOI:** 10.1259/bjrcr.20150100

**Published:** 2015-07-03

**Authors:** M A Jacobs, C Lanciault, S Weinstein

**Affiliations:** ^1^Department of Radiology, Oregon Health and Science University, Portland, OR, USA; ^2^Vision Radiology, Pittsburgh, PA, USA; ^3^Department of Pathology, Oregon Health and Science University, Portland, OR, USA; ^4^Department of Radiology, UCSF Medical Center, San Francisco, CA, USA; ^5^San Francisco VA Medical Center, San Francisco, CA, USA

## Abstract

We present a case of an incidentally detected cystic liver mass on CT scan, with histology showing biliary epithelium embedded in fibrous stroma and dysplastic features, consistent with an adenofibroma. This is only the third case described in the literature with malignant histology and the first case with angiographic imaging and subsequent management with preoperative embolization prior to surgical resection. We discuss the differential of the imaging findings and the features of this rare entity. Because of the risk of malignant transformation, consideration of this tumour is important to ensure early detection and ultimately resection for improved survival.

## Clinical presentation

We report a case of a 57-year-old female who was incidentally found to have a large hepatic mass on a CT scan performed for left flank and lower abdominal pain for 5 days. The patient had no history of jaundice, hepatitis or alcohol abuse. Her medical history included a prior blood transfusion.

On clinical exam, the patient demonstrated modest left costovertebral angle tenderness. Her abdomen was soft, but she was tender in the left lower quadrant. Bowel sounds were somewhat decreased in the left lower abdomen. Laboratory data revealed a mild leukocytosis, and she was presumed to have diverticulitis and was treated with ciprofloxacin and metronidazole. Her symptoms worsened, and she presented to the emergency department the following day. At that time, her laboratory values had returned to normal.

Laboratory work-up included liver enzymes, which were normal, as were her carcinoembryonic antigen and α-fetoprotein. Colonoscopy was also performed and was negative for malignancy.

## Imaging findings/investigations

**Figure 1.**
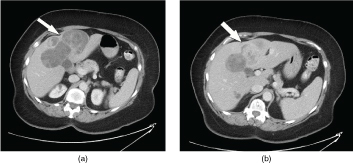
(a,b) Multiple axial CT images with contrast through the liver demonstrate a heterogeneous, predominately cystic, partially subcapsular mass (white arrows) centred in the anterior segment of the right hepatic lobe.

A CT scan of the abdomen showed an incidental large, 11.8×7.5 cm**,** lobulated, heterogeneous, predominantly hypodense mass with irregular internal areas of enhancement. The mass was centred in the anterior segment of the right hepatic lobe, extending into the medial segment of the left hepatic lobe and deforming the liver contour (Figure 1a,b). An indeterminate similar-appearing satellite lesion was present in hepatic segment 6. No evidence of metastatic disease was present. Mild diverticulitis was noted in the left lower quadrant (not shown).

Differential diagnosis of the imaging findings would include rare benign biliary neoplasms, including bile duct adenoma, biliary cystadenoma, a congenital biliary cyst with superimposed complication such as haemorrhage or infection or, less likely, a von Meyenburg complex.^1^ Included in the differential would be malignant lesions such as biliary cystadenocarcinoma, cholangiocarcinoma or cystic metastasis and an atypical abscess.

Abscess was put at the top of the differential diagnosis at that time and an attempt was made to drain it under CT guidance. As no material could be aspirated, biopsy specimens were subsequently obtained under CT guidance with a 16-gauge needle targeting the more solid component.

**Figure 2.**
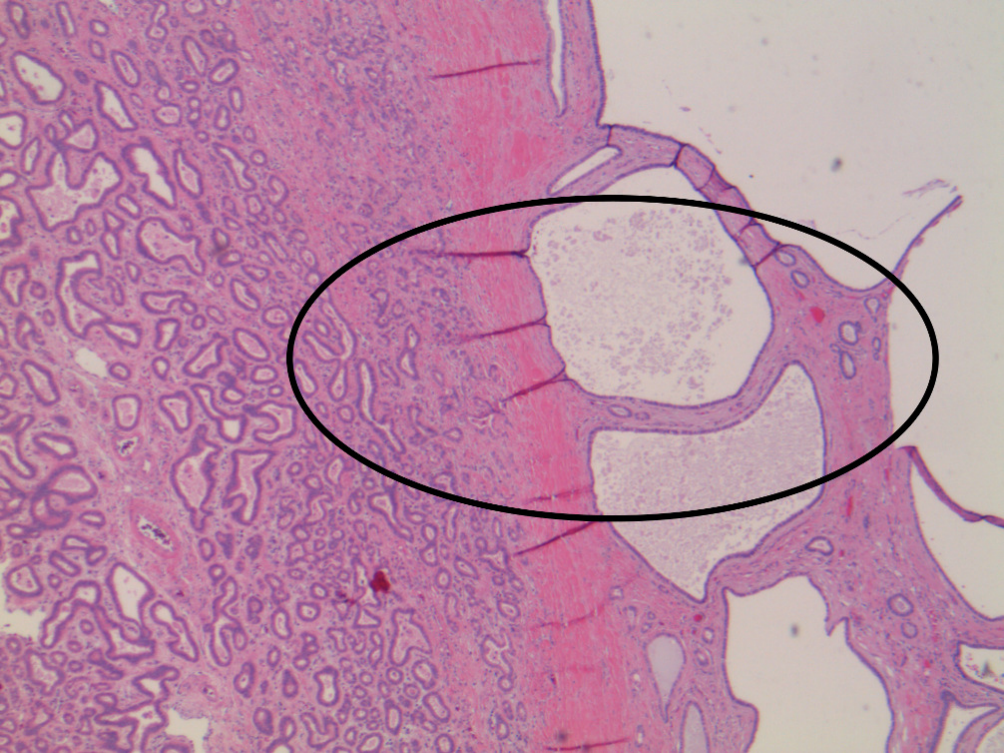
There are cystic, microcystic and tubular structures lined by cuboidal biliary epithelium embedded in fibrous stroma consistent with biliary adenofibroma. The fibroepithelial proliferation within the larger cystic spaces (circle) is characteristic of this entity.

**Figure 3.**
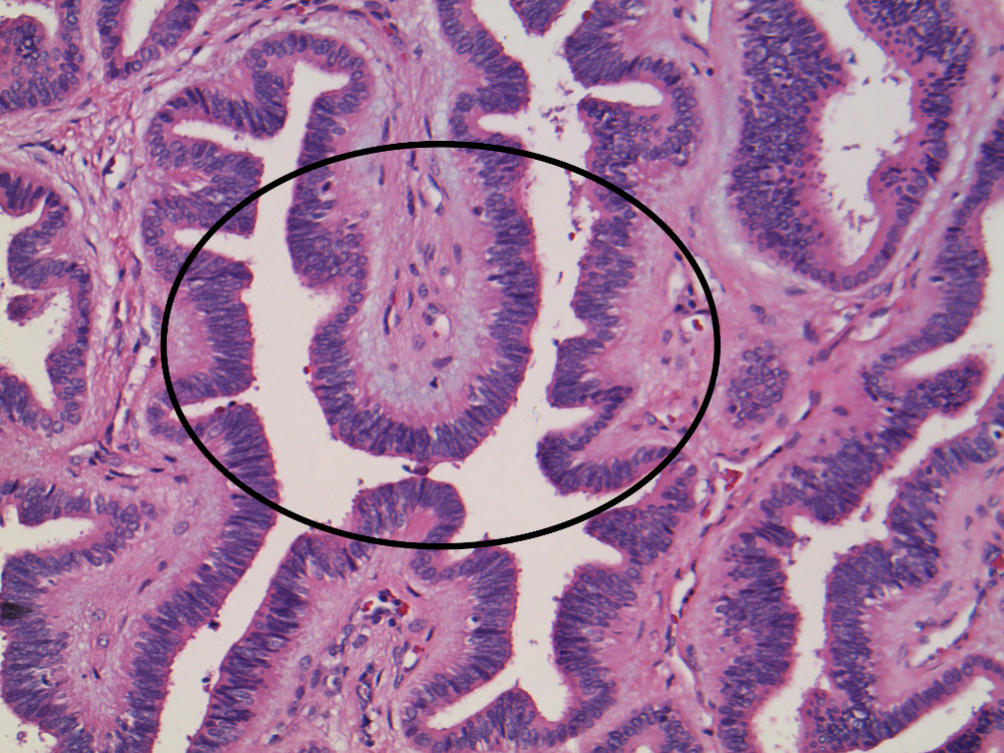
An area of low-grade dysplasia is noted with more columnar-type epithelium with elongated, hyperchromatic nuclei, nuclear crowding and pseudopallisading (circle).

Surgical resection was recommended. Histology revealed biliary adenofibroma (BAF) with dysplasia and architectural features concerning for malignant potential (Figures 2 and 3).

## Histological findings

Right trisegmentectomy gross specimen revealed a 10 × 9.5 × 7.5-cm well-circumscribed mass comprising serous-filled cysts, multiloculated areas with spongy pink areas and fleshy-to-firm white–pink areas. There was a second lesion identical in appearance. Histologically, there were cystic, microcystic and tubular structures lined by cuboidal biliary epithelium embedded in fibrous stroma consistent with BAF. The fibroepithelial proliferation within the larger cystic spaces is characteristic of this entity (Figure 2). There was no evidence of invasion. An area of low-grade dysplasia showing more columnar-type epithelium with elongated, hyperchromatic nuclei was identified. There was nuclear crowding and pseudopallisading (Figure 3). In contrast, bile duct adenomas or peribiliary hamartomas tend to be much smaller and would not exhibit the cystic dilation as seen in this case. The gland spacing would be tighter with less stroma and less cystic configurations.^2^

## Management/treatment

**Figure 4.**
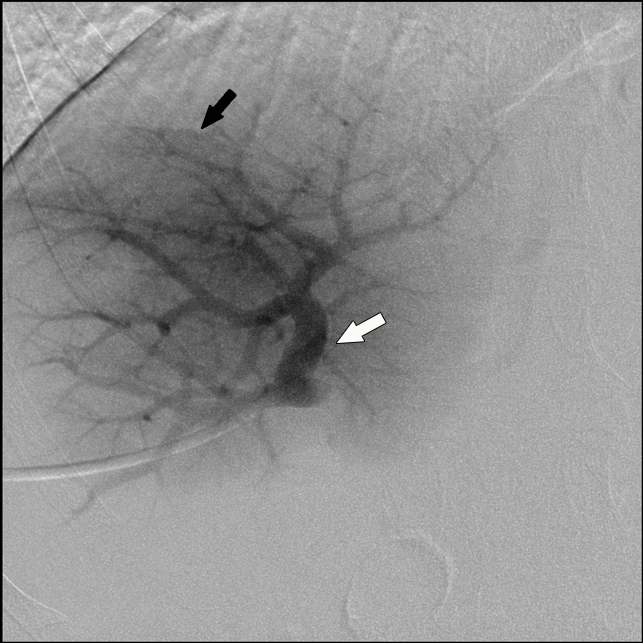
Portal venography image demonstrates right portal vein branches (white arrow) that are moderately displaced and distorted by the mass that is faintly enhancing (black arrow).

Portal venography pre-embolization showed normal-appearing lateral segment left portal vein (not shown). The medial left and anterior right portal vein branches were moderately displaced by the mass with external compression (Figure 4). Preoperatively, a right portal vein and segment 4 portal vein inflow embolization with particles and coils was performed to maximize contralateral hepatic lobe hypertrophy. The patient subsequently underwent a resection of the right hepatic lobe and medial segment of the left hepatic lobe. The patient has undergone yearly abdominal examinations with CT scans for surveillance without evidence of recurrence for 5 years.

## Discussion

We report the imaging and pathological findings and management of a rare case of BAF of the liver. Based on dysplastic histological findings, this lesion was managed surgically. This is only the third case of reported malignant features and the first case treated with preoperative portal vein embolization.

BAF was first described in 1993 by Tsui et al^3^; 7 cases of BAF have been reported in the pathology and gastroenterology literature, more commonly in women than men, with malignant progression/transformation documented in only 2 cases.^1,3–10^ The aetiology is unknown but the immunophenotypic profile may suggest a bile or interlobular duct origin. Intrahepatic bile ducts develop from ductal plate embryologically. Development disorders of the ductal plates are thought to make up a large proportion of congenital disease of the intrahepatic bile ducts, also known as fibrocystic liver disease resulting from abnormal embryogenesis of the biliary tree.

Patients may present with pain in the right upper quadrant from mass effect of the lesion. The location of this patient’s pain was atypical. Imaging findings have demonstrated a large subcapsular mass, occasionally multiple, in either cirrhotic or normal livers. Imaging features are non-specific, however, and biopsy is required. The role of biopsy in cystic lesions is debatable. If possible, it is advisable to target the wall or a nodular component to improve diagnostic yield. Yield can be lower in cystic or necrotic masses and give a false-negative cytological diagnosis because the aspirate is less cellular, with mostly macrophages.^11^ There is a theoretical risk of seeding the tract. Fine needle aspiration cannot serve as an exclusive diagnostic method for malignant lesions because of false negatives. Improved diagnostic rate is obtained with cytology and histology. The risk of seeding the tract has been reported with an incidence of less than 0.009%.^12^ On histology, there is a resemblance to biliary hamartomas and bile duct adenomas. The large size would be atypical for biliary hamartoma. Nuclear accumulation of p53 and increased mitotic activity has been reported with premalignant lesions. Infiltration in the liver and the presence of metastasis are features suggestive of frank malignancy.^6^

Preoperative embolization is used to induce compensatory hypertrophy of the non-involved liver segment by causing an ischaemia in the area of the liver that will subsequently be removed by occluding its blood supply. Because the liver is able to tolerate a large resection and regenerate to ample size, portal vein embolization can lead to an increase in volume in the non-targeted liver segments.^13^ Because of the dual bloody supply, portal vein embolization does not cause complications associated with liver necrosis. Portal vein embolization has been performed successfully for liver and metastatic liver tumours. Various materials have been used for portal vein occlusion.^14^ Clinical management of this disease, including a recommended surveillance interval, has not been defined due to its rarity.

## Learning points

1. BAF is a very rare tumour of the liver.
2. This lesion should be considered in patients with unusual complex cystic and solid masses of the liver.
3. Malignant transformation can occur and early recognition, biopsy and, if indicated, resection, may improve survival.
